# (3-Hydroxy-2-{[1-(2-oxidophenyl)ethyl­idene]amino-κ^2^ *O*,*N*}propanoato-κ*O* ^1^)diphenyltin(IV)

**DOI:** 10.1107/S1600536810039449

**Published:** 2010-10-09

**Authors:** Yan Qiao, Xiuping Ju, Zhiqing Gao, Lingqian Kong

**Affiliations:** a Dongchang College, Liaocheng University, Liaocheng, 250059, People’s Republic of China

## Abstract

In the title compound, [Sn(C_6_H_5_)_2_(C_11_H_11_NO_4_)], the tin(IV) atom is penta-coordinated in a distorted trigonal-bipyramidal SnC_2_NO_2_ geometry. In the crystal structure, inter­molecular O—H⋯O hydrogen bonds link the mol­ecules into centrosymmetric dimers. Weak C—H⋯O inter­actions further link the dimers into chains extending in [010].

## Related literature

For applications and biological activity of organotin compounds, see: Chandrasekhar *et al.* (2008 ▶); Collinson & Fenton (1996 ▶). For related structures, see: Beltran *et al.* (2003 ▶); Tian *et al.* (2004 ▶).
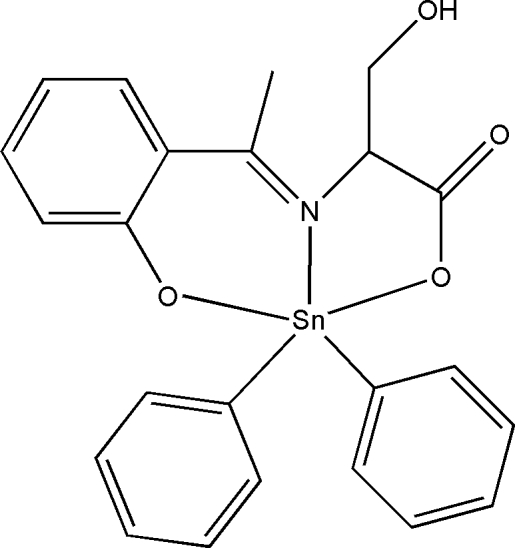


## Experimental

### 

#### Crystal data

[Sn(C_6_H_5_)_2_(C_11_H_11_NO_4_)]
*M* *_r_* = 494.10
Monoclinic, 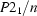

*a* = 11.234 (10) Å
*b* = 15.581 (14) Å
*c* = 12.321 (11) Å
β = 111.488 (12)°
*V* = 2007 (3) Å^3^
*Z* = 4
Mo *K*α radiation
μ = 1.30 mm^−1^
*T* = 298 K
0.48 × 0.45 × 0.19 mm

#### Data collection

Bruker SMART APEX CCD area-detector diffractometer
Absorption correction: multi-scan (*SADABS*; Sheldrick, 1996 ▶) *T*_min_ = 0.574, *T*_max_ = 0.790
10058 measured reflections
3526 independent reflections
2572 reflections with *I* > 2σ(*I*)
*R*_int_ = 0.065

#### Refinement

*R*[*F* ^2^ > 2σ(*F* ^2^)] = 0.048
*wR*(*F* ^2^) = 0.128
*S* = 1.00
3526 reflections
264 parameters
H-atom parameters constrained
Δρ_max_ = 1.48 e Å^−3^
Δρ_min_ = −0.80 e Å^−3^

### 

Data collection: *SMART* (Bruker, 2007 ▶); cell refinement: *SAINT* (Bruker, 2007 ▶); data reduction: *SAINT*; program(s) used to solve structure: *SHELXS97* (Sheldrick, 2008 ▶); program(s) used to refine structure: *SHELXL97* (Sheldrick, 2008 ▶); molecular graphics: *SHELXTL* (Sheldrick, 2008 ▶); software used to prepare material for publication: *SHELXTL*.

## Supplementary Material

Crystal structure: contains datablocks I, global. DOI: 10.1107/S1600536810039449/cv2769sup1.cif

Structure factors: contains datablocks I. DOI: 10.1107/S1600536810039449/cv2769Isup2.hkl

Additional supplementary materials:  crystallographic information; 3D view; checkCIF report

## Figures and Tables

**Table 1:** Hydrogen-bond geometry (Å, °)

*D*—H⋯*A*	*D*—H	H⋯*A*	*D*⋯*A*	*D*—H⋯*A*
C19—H19⋯O2^i^	0.93	2.43	3.215 (8)	143
O3—H3⋯O4^ii^	0.82	2.00	2.760 (6)	153

Symmetry codes: (i) 
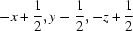
; (ii) 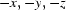.

**Fig. 1.**
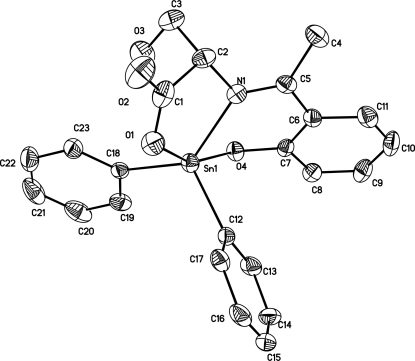
A view of (I) showing the atomic numbering scheme and 30% probability displacement ellipsoids. H atoms omitted for clarity.

## supplementary crystallographic information

### Comment

Organotin compounds are of current interest due to their industrial, agricultural and biological applications (Chandrasekhar *et al.*, 2008). Meanwhile, the chemistry of organotin(IV) complexes of Schiff bases has stemmed from the reported biocidal and anti-tumor activities of organotin(IV) complexes and the behavior of Schiff bases as models for biological systems (Collinson *et al.*, 1996). As a contribution to this field of science, we report here the crystal structure of the title compound, (I).

In (I) (Fig. 1), the bond lengths and angles are normal and comparable to those observed in the similar compounds (Beltran *et al.*, 2003; Tian *et al.*, 2004). The Sn1 atom has distorted trigonal-bipyramidal environment, with atoms O1 and O4 in axial positions [O1—Sn1—O4  156.40 (15) °], and the C12 and C18 atoms of two phenyl groups and the imino N1 atom in equatorial positions. Associated with the sum of the angles subtended at the Sn1 in the equatorial plane is 359.7 (4) °, indicating approximate coplanarity for these atoms. The coordinate Sn—O bond lengths of 2.129 (4) and  2.126 (5) Å, respectively, are close to those observed in the reported organotin compounds (Beltran *et al.*, 2003;Tian *et al.*, 2004).

Intermolecular O—H···O hydrogen bonds (Table 1) link the molecules into centrosymmetric dimers in the crystal structure, and weak C—H···O interactions (Table 1) link further these dimers into chains extended in direction [010].

### Experimental

Diphenyltin chloride (3 mmol), *L*-Serine (3 mmol) and 2-hydroxyacetophenone 3 mmol) in 20 ml of benzene were refluxed for 24 h. The resulting clear solution was evaporated under vacuum and the colorless crystalline material obtained was recrystallized from methanol. The product was then dissolved in dichloromethane-hexane, and colourless crystals were grown by slow evaporation.

### Refinement

All H atoms were placed in geometrically idealized positions (O—H = 0.82 Å, C—H = 0.93 - 0.97 Å) and treated as riding on their parent atoms, with *U*_iso_(H) = 1.2–1.5 *U*_eq_(C, O) (C,*O*).

### Crystal data

**Table:**

[Sn(C_6_H_5_)_2_(C_11_H_11_NO_4_)]	*F*(000) = 992
*M**_r_* = 494.10	*D*_x_ = 1.635 Mg m^−^^3^
Monoclinic, *P*2_1_/*n*	Mo *K*α radiation, λ = 0.71073 Å
Hall symbol: -P 2yn	Cell parameters from 3469 reflections
*a* = 11.234 (10) Å	θ = 2.2–25.6°
*b* = 15.581 (14) Å	µ = 1.30 mm^−^^1^
*c* = 12.321 (11) Å	*T* = 298 K
β = 111.488 (12)°	Block, colourless
*V* = 2007 (3) Å^3^	0.48 × 0.45 × 0.19 mm
*Z* = 4	

### Data collection

**Table:**

Bruker SMART APEX CCD area-detector diffractometer	3526 independent reflections
Radiation source: fine-focus sealed tube	2572 reflections with *I* > 2σ(*I*)
graphite	*R*_int_ = 0.065
phi and ω scans	θ_max_ = 25.0°, θ_min_ = 2.2°
Absorption correction: multi-scan (*SADABS*; Sheldrick, 1996)	*h* = −13→12
*T*_min_ = 0.574, *T*_max_ = 0.790	*k* = −18→18
10058 measured reflections	*l* = −14→13

### Refinement

**Table:**

Refinement on *F*^2^	Primary atom site location: structure-invariant direct methods
Least-squares matrix: full	Secondary atom site location: difference Fourier map
*R*[*F*^2^ > 2σ(*F*^2^)] = 0.048	Hydrogen site location: inferred from neighbouring sites
*wR*(*F*^2^) = 0.128	H-atom parameters constrained
*S* = 1.00	*w* = 1/[σ^2^(*F*_o_^2^) + (0.0704*P*)^2^] where *P* = (*F*_o_^2^ + 2*F*_c_^2^)/3
3526 reflections	(Δ/σ)_max_ = 0.001
264 parameters	Δρ_max_ = 1.48 e Å^−^^3^
0 restraints	Δρ_min_ = −0.80 e Å^−^^3^

### Special details

**Table:**

Geometry. All e.s.d.'s (except the e.s.d. in the dihedral angle between two l.s. planes) are estimated using the full covariance matrix. The cell e.s.d.'s are taken into account individually in the estimation of e.s.d.'s in distances, angles and torsion angles; correlations between e.s.d.'s in cell parameters are only used when they are defined by crystal symmetry. An approximate (isotropic) treatment of cell e.s.d.'s is used for estimating e.s.d.'s involving l.s. planes.
Refinement. Refinement of *F*^2^ against ALL reflections. The weighted *R*-factor *wR* and goodness of fit *S* are based on *F*^2^, conventional *R*-factors *R* are based on *F*, with *F* set to zero for negative *F*^2^. The threshold expression of *F*^2^ > σ(*F*^2^) is used only for calculating *R*-factors(gt) *etc*. and is not relevant to the choice of reflections for refinement. *R*-factors based on *F*^2^ are statistically about twice as large as those based on *F*, and *R*- factors based on ALL data will be even larger.

### Fractional atomic coordinates and isotropic or equivalent isotropic displacement parameters (Å^2^)

**Table:**

	*x*	*y*	*z*	*U*_iso_*/*U*_eq_	
Sn1	0.24581 (4)	0.04875 (2)	0.16787 (3)	0.03717 (17)	
N1	0.2654 (4)	0.0895 (3)	0.0054 (4)	0.0386 (10)	
O1	0.3025 (4)	0.1798 (2)	0.1969 (4)	0.0509 (10)	
O2	0.3143 (4)	0.3031 (2)	0.1110 (4)	0.0736 (14)	
O3	0.0413 (4)	0.1585 (3)	−0.0006 (4)	0.0699 (13)	
H3	−0.0185	0.1283	−0.0408	0.105*	
O4	0.1812 (3)	−0.0632 (2)	0.0708 (3)	0.0399 (9)	
C1	0.2928 (5)	0.2269 (3)	0.1086 (6)	0.0500 (15)	
C2	0.2441 (5)	0.1820 (3)	−0.0101 (5)	0.0437 (13)	
H2	0.2898	0.2044	−0.0582	0.052*	
C3	0.1007 (6)	0.1969 (4)	−0.0723 (6)	0.0582 (17)	
H3A	0.0820	0.2579	−0.0812	0.070*	
H3B	0.0707	0.1706	−0.1489	0.070*	
C4	0.3215 (6)	0.0810 (4)	−0.1666 (5)	0.0556 (16)	
H4A	0.2582	0.1245	−0.2009	0.083*	
H4B	0.3158	0.0376	−0.2236	0.083*	
H4C	0.4051	0.1065	−0.1402	0.083*	
C5	0.2986 (5)	0.0412 (3)	−0.0648 (5)	0.0394 (13)	
C6	0.3123 (5)	−0.0518 (3)	−0.0481 (5)	0.0381 (13)	
C7	0.2531 (5)	−0.0993 (3)	0.0162 (5)	0.0364 (12)	
C8	0.2663 (5)	−0.1884 (3)	0.0221 (5)	0.0449 (14)	
H8	0.2268	−0.2199	0.0636	0.054*	
C9	0.3374 (6)	−0.2310 (4)	−0.0330 (6)	0.0559 (16)	
H9	0.3451	−0.2904	−0.0279	0.067*	
C10	0.3962 (6)	−0.1856 (4)	−0.0949 (6)	0.0570 (17)	
H10	0.4438	−0.2142	−0.1316	0.068*	
C11	0.3844 (6)	−0.0978 (4)	−0.1022 (5)	0.0509 (15)	
H11	0.4249	−0.0676	−0.1439	0.061*	
C12	0.4214 (5)	−0.0027 (3)	0.2843 (4)	0.0358 (12)	
C13	0.4355 (6)	−0.0908 (3)	0.3081 (5)	0.0458 (14)	
H13	0.3706	−0.1285	0.2664	0.055*	
C14	0.5452 (6)	−0.1222 (4)	0.3931 (5)	0.0522 (16)	
H14	0.5535	−0.1808	0.4082	0.063*	
C15	0.6420 (6)	−0.0677 (4)	0.4556 (5)	0.0552 (16)	
H15	0.7161	−0.0896	0.5116	0.066*	
C16	0.6298 (6)	0.0187 (4)	0.4356 (5)	0.0523 (16)	
H16	0.6948	0.0556	0.4790	0.063*	
C17	0.5208 (6)	0.0511 (3)	0.3509 (5)	0.0424 (14)	
H17	0.5135	0.1100	0.3378	0.051*	
C18	0.0980 (5)	0.0592 (3)	0.2353 (5)	0.0360 (12)	
C19	0.0678 (6)	−0.0131 (4)	0.2858 (5)	0.0513 (15)	
H19	0.1058	−0.0654	0.2814	0.062*	
C20	−0.0187 (7)	−0.0087 (6)	0.3429 (6)	0.074 (2)	
H20	−0.0386	−0.0575	0.3763	0.089*	
C21	−0.0740 (7)	0.0690 (7)	0.3492 (7)	0.083 (3)	
H21	−0.1310	0.0728	0.3878	0.099*	
C22	−0.0456 (6)	0.1416 (5)	0.2987 (6)	0.070 (2)	
H22	−0.0847	0.1936	0.3020	0.083*	
C23	0.0414 (6)	0.1364 (4)	0.2432 (5)	0.0503 (15)	
H23	0.0620	0.1855	0.2108	0.060*	

### Atomic displacement parameters (Å^2^)

**Table:**

	*U*^11^	*U*^22^	*U*^33^	*U*^12^	*U*^13^	*U*^23^
Sn1	0.0371 (3)	0.0369 (2)	0.0355 (3)	−0.00405 (16)	0.01101 (19)	−0.00707 (15)
N1	0.036 (3)	0.037 (2)	0.040 (3)	−0.002 (2)	0.011 (2)	−0.001 (2)
O1	0.053 (3)	0.042 (2)	0.053 (3)	−0.0069 (19)	0.014 (2)	−0.0080 (18)
O2	0.079 (3)	0.034 (2)	0.101 (4)	−0.008 (2)	0.025 (3)	−0.010 (2)
O3	0.043 (3)	0.104 (4)	0.057 (3)	−0.017 (2)	0.013 (2)	−0.015 (3)
O4	0.035 (2)	0.0420 (19)	0.041 (2)	−0.0073 (16)	0.0123 (19)	−0.0076 (16)
C1	0.033 (3)	0.042 (3)	0.073 (5)	−0.005 (3)	0.016 (3)	−0.012 (3)
C2	0.038 (3)	0.040 (3)	0.048 (4)	−0.002 (2)	0.010 (3)	0.008 (3)
C3	0.058 (4)	0.045 (3)	0.064 (5)	0.004 (3)	0.014 (4)	0.012 (3)
C4	0.058 (4)	0.068 (4)	0.043 (4)	−0.005 (3)	0.020 (3)	−0.001 (3)
C5	0.028 (3)	0.052 (3)	0.032 (3)	−0.007 (2)	0.004 (2)	−0.001 (2)
C6	0.034 (3)	0.045 (3)	0.029 (3)	−0.004 (2)	0.006 (3)	−0.010 (2)
C7	0.027 (3)	0.039 (3)	0.038 (3)	0.002 (2)	0.006 (2)	−0.009 (2)
C8	0.041 (4)	0.045 (3)	0.043 (4)	−0.003 (3)	0.008 (3)	−0.004 (2)
C9	0.044 (4)	0.047 (3)	0.071 (5)	0.003 (3)	0.015 (3)	−0.019 (3)
C10	0.042 (4)	0.064 (4)	0.064 (5)	0.005 (3)	0.018 (3)	−0.024 (3)
C11	0.042 (4)	0.066 (4)	0.042 (4)	−0.007 (3)	0.013 (3)	−0.009 (3)
C12	0.027 (3)	0.048 (3)	0.029 (3)	0.001 (2)	0.006 (2)	−0.009 (2)
C13	0.051 (4)	0.044 (3)	0.033 (3)	−0.009 (3)	0.006 (3)	−0.011 (2)
C14	0.054 (4)	0.056 (3)	0.033 (3)	0.012 (3)	0.000 (3)	0.000 (3)
C15	0.045 (4)	0.084 (5)	0.030 (3)	0.017 (3)	0.005 (3)	−0.004 (3)
C16	0.042 (4)	0.082 (4)	0.030 (3)	−0.012 (3)	0.010 (3)	−0.020 (3)
C17	0.044 (4)	0.047 (3)	0.039 (3)	−0.006 (3)	0.018 (3)	−0.010 (2)
C18	0.029 (3)	0.046 (3)	0.029 (3)	0.000 (2)	0.006 (2)	−0.002 (2)
C19	0.043 (4)	0.066 (4)	0.037 (4)	−0.004 (3)	0.005 (3)	0.002 (3)
C20	0.063 (5)	0.113 (6)	0.047 (4)	−0.025 (5)	0.021 (4)	0.005 (4)
C21	0.046 (5)	0.156 (8)	0.053 (5)	−0.006 (5)	0.026 (4)	−0.025 (5)
C22	0.049 (4)	0.094 (5)	0.061 (5)	0.016 (4)	0.014 (4)	−0.023 (4)
C23	0.048 (4)	0.058 (3)	0.041 (4)	0.007 (3)	0.011 (3)	−0.009 (3)

### Geometric parameters (Å, °)

**Table:**

Sn1—O4	2.089 (3)	C9—C10	1.374 (8)
Sn1—C18	2.118 (5)	C9—H9	0.9300
Sn1—C12	2.126 (5)	C10—C11	1.375 (8)
Sn1—O1	2.129 (4)	C10—H10	0.9300
Sn1—N1	2.187 (5)	C11—H11	0.9300
N1—C5	1.299 (6)	C12—C13	1.401 (8)
N1—C2	1.463 (6)	C12—C17	1.398 (7)
O1—C1	1.283 (7)	C13—C14	1.381 (8)
O2—C1	1.210 (6)	C13—H13	0.9300
O3—C3	1.420 (7)	C14—C15	1.372 (8)
O3—H3	0.8200	C14—H14	0.9300
O4—C7	1.349 (6)	C15—C16	1.366 (8)
C1—C2	1.530 (8)	C15—H15	0.9300
C2—C3	1.527 (8)	C16—C17	1.381 (9)
C2—H2	0.9800	C16—H16	0.9300
C3—H3A	0.9700	C17—H17	0.9300
C3—H3B	0.9700	C18—C23	1.381 (7)
C4—C5	1.504 (8)	C18—C19	1.387 (7)
C4—H4A	0.9600	C19—C20	1.395 (9)
C4—H4B	0.9600	C19—H19	0.9300
C4—H4C	0.9600	C20—C21	1.375 (11)
C5—C6	1.464 (7)	C20—H20	0.9300
C6—C7	1.415 (7)	C21—C22	1.382 (10)
C6—C11	1.418 (7)	C21—H21	0.9300
C7—C8	1.395 (7)	C22—C23	1.386 (8)
C8—C9	1.391 (7)	C22—H22	0.9300
C8—H8	0.9300	C23—H23	0.9300
			
O4—Sn1—C18	97.52 (17)	C9—C8—H8	119.4
O4—Sn1—C12	96.51 (17)	C7—C8—H8	119.4
C18—Sn1—C12	115.5 (2)	C10—C9—C8	120.3 (5)
O4—Sn1—O1	156.40 (15)	C10—C9—H9	119.8
C18—Sn1—O1	95.16 (17)	C8—C9—H9	119.8
C12—Sn1—O1	95.85 (18)	C9—C10—C11	119.5 (5)
O4—Sn1—N1	81.29 (15)	C9—C10—H10	120.2
C18—Sn1—N1	133.90 (18)	C11—C10—H10	120.2
C12—Sn1—N1	110.33 (18)	C10—C11—C6	122.1 (6)
O1—Sn1—N1	75.58 (15)	C10—C11—H11	119.0
C5—N1—C2	123.9 (5)	C6—C11—H11	119.0
C5—N1—Sn1	126.2 (3)	C13—C12—C17	117.4 (5)
C2—N1—Sn1	109.8 (3)	C13—C12—Sn1	121.1 (4)
C1—O1—Sn1	118.6 (4)	C17—C12—Sn1	121.0 (4)
C3—O3—H3	109.5	C14—C13—C12	120.4 (5)
C7—O4—Sn1	119.2 (3)	C14—C13—H13	119.8
O2—C1—O1	125.9 (6)	C12—C13—H13	119.8
O2—C1—C2	118.0 (6)	C15—C14—C13	120.6 (5)
O1—C1—C2	116.0 (5)	C15—C14—H14	119.7
N1—C2—C1	110.0 (5)	C13—C14—H14	119.7
N1—C2—C3	107.9 (4)	C16—C15—C14	120.1 (6)
C1—C2—C3	110.6 (5)	C16—C15—H15	119.9
N1—C2—H2	109.4	C14—C15—H15	119.9
C1—C2—H2	109.4	C15—C16—C17	120.0 (6)
C3—C2—H2	109.4	C15—C16—H16	120.0
O3—C3—C2	105.9 (5)	C17—C16—H16	120.0
O3—C3—H3A	110.6	C16—C17—C12	121.3 (5)
C2—C3—H3A	110.6	C16—C17—H17	119.3
O3—C3—H3B	110.6	C12—C17—H17	119.3
C2—C3—H3B	110.6	C23—C18—C19	118.8 (5)
H3A—C3—H3B	108.7	C23—C18—Sn1	122.8 (4)
C5—C4—H4A	109.5	C19—C18—Sn1	118.0 (4)
C5—C4—H4B	109.5	C18—C19—C20	121.1 (6)
H4A—C4—H4B	109.5	C18—C19—H19	119.5
C5—C4—H4C	109.5	C20—C19—H19	119.5
H4A—C4—H4C	109.5	C21—C20—C19	119.0 (7)
H4B—C4—H4C	109.5	C21—C20—H20	120.5
N1—C5—C6	121.3 (5)	C19—C20—H20	120.5
N1—C5—C4	119.7 (5)	C20—C21—C22	120.7 (7)
C6—C5—C4	119.0 (5)	C20—C21—H21	119.6
C7—C6—C11	117.7 (5)	C22—C21—H21	119.6
C7—C6—C5	123.4 (5)	C21—C22—C23	119.7 (6)
C11—C6—C5	118.8 (5)	C21—C22—H22	120.1
O4—C7—C8	117.4 (5)	C23—C22—H22	120.1
O4—C7—C6	123.4 (4)	C18—C23—C22	120.7 (6)
C8—C7—C6	119.2 (5)	C18—C23—H23	119.6
C9—C8—C7	121.2 (5)	C22—C23—H23	119.6

### Hydrogen-bond geometry (Å, °)

**Table:**

*D*—H···*A*	*D*—H	H···*A*	*D*···*A*	*D*—H···*A*
C19—H19···O2^i^	0.93	2.43	3.215 (8)	143
O3—H3···O4^ii^	0.82	2.00	2.760 (6)	153

Symmetry codes: (i) −*x*+1/2, *y*−1/2, −*z*+1/2; (ii) −*x*, −*y*, −*z*.
